# Timing of Return to Driving After Volar Locking–Plate Fixation of Distal Radius Fractures Without Wrist Immobilization: A Prospective Driving Simulator Study

**DOI:** 10.2106/JBJS.OA.26.00013

**Published:** 2026-07-02

**Authors:** Rikiya Shirato, Yuka Yamanaka, Wataru Goshima, Takashi Oda, Takuro Wada

**Affiliations:** 1 Department of Rehabilitation, Faculty of Healthcare and Science, Hokkaido Bunkyo University, Eniwa, Japan; 2 Department of Rehabilitation, Saiseikai Otaru Hospital, Otaru, Japan; 3 Department of Orthopaedic Surgery, Saiseikai Otaru Hospital, Otaru, Japan

## Abstract

**Background:**

Studies examining return to driving after volar locking–plate fixation (VLP) for distal radius fractures (DRFs) have used heterogeneous postoperative immobilization protocols, resulting in widely varying recommendations for the timing of return to driving. The aim of this study was to determine the timing of return to driving in patients with DRFs treated with VLP managed without postoperative immobilization.

**Methods:**

Patients with DRFs treated with VLP and healthy controls who routinely drive were prospectively enrolled. Driving performance was evaluated at postoperative weeks 1, 2, 3, 4, 6, and 8 using a driving simulator (DS), administered as 2 tasks: the Steering Operation Test (pylon-passing) and the Hazard Anticipation Task (urban driving). The Steering Operation Test outcomes included reaction time, accuracy, left-right balance, and adaptability. The Hazard Anticipation Task outcomes included maximum steering angle, number of collisions, percentage of distance speeding, average excess speed, average turning speed at intersections, and number of turn-signal errors. Moreover, demographic and clinical factors potentially associated with driving performance were explored only among patients with DRFs using multivariable analysis.

**Results:**

Twenty-seven patients completed the DS assessment at all 6 point sessions, and 25 healthy controls completed a single session. Accuracy on the Steering Operation Test was significantly lower in patients than in controls at postoperative weeks 1 and 2, whereas no significant between-group differences were observed from week 3 onward. No significant differences were observed between the groups in any of the indicators for the Hazard Anticipation Task. Multivariable analysis showed a negative correlation of steering accuracy with female sex and older age and a positive correlation with forearm pronation.

**Conclusions:**

In patients with DRFs after VLP without wrist immobilization, driving performance did not differ significantly from that of healthy controls at postoperative week 3. These findings suggest that postoperative week 3 could serve as a reference point when considering return to driving. Gradual and careful guidance on returning to driving is recommended for women, older adults, and patients with insufficient recovery in forearm pronation.

**Level of Evidence:**

Prognostic Level III. See Instructions for Authors for a complete description of levels of evidence.

## Introduction

Distal radius fractures (DRFs) are the most common fractures, representing 16.4% of all fractures^1^. Volar locking–plate fixation (VLP) is widely used as a standard operative treatment of DRFs^2^. During the early postoperative period following VLP, wrist dysfunction is substantial^3^, directly affecting driving operations. Therefore, during follow-up visits, patients commonly ask when they may safely resume driving^4,5^.

Safe driving requires the coordinated integration of cognitive and neural systems, as well as sufficient upper-limb and lower-limb range of motion (ROM) to perform key tasks such as steering and braking^6,7^. Steering, defined as limb movement used to rotate the steering wheel and control vehicle direction, is the primary upper-extremity task involved in driving^8^.

Patients with musculoskeletal disabilities have a much higher risk of at-fault accidents^9^. In addition, 36% of patients with musculoskeletal disorders resume driving without consulting a physician, and 19% feel unsafe to drive^10^. Therefore, patient-initiated return to driving based on self-judgment is hazardous, underscoring the need for evidence-based recommendations on when driving can be safely resumed. Although driving fitness is typically determined by the treating physician^11,12^, objective evidence and clear guidelines to support such decisions are limited^11,13^. Consequently, clinical recommendations vary widely, ranging from allowing immediate driving to restricting driving for up to 12 weeks^5^.

In recent years, few studies have evaluated the timing of return to driving after VLP for DRFs and suggested intervals vary from 3 to 8 weeks^4,14,15^. This variation likely reflects differences in assessment methods, such as the use of a driving simulator (DS) vs. an on-road vehicle test, as well as differences in outcome measures and testing conditions, including the duration of postoperative immobilization and whether driving performance was assessed with or without immobilization. Wrist immobilization reportedly affects both subjective and objective driving performance^16,17^.

More recently, early wrist mobilization without external immobilization has been shown to promote functional recovery^18-20^. Accordingly, we longitudinally evaluated driving performance in patients with DRFs who underwent early mobilization without external immobilization after VLP using a DS and compared their performance with that of healthy controls. We aimed to identify the postoperative time point at which the driving performance of patients no longer differed significantly from that of healthy controls, potentially serving as a reference point when considering return to driving. In addition, we aimed to identify factors associated with driving performance recovery. We hypothesized that return to driving would be possible at an earlier point than those reported in previous studies.

## Materials and Methods

### Participants

From January 2021 to April 2025, consecutive patients aged 20 to 80 years who underwent VLP for a DRF at a single institution were invited to participate in this prospective cohort study. Written informed consent was obtained from all participants; the study was approved by the institutional review boards of our university and the affiliated hospital (No. 03004 and R3-7). We included patients with unilateral, isolated DRFs who had a valid driver’s license and drove at least once per week. We excluded participants with concomitant or prior upper-extremity trauma involving the operative and/or nonoperative limb, concomitant injuries of the upper or lower extremities that could affect steering control or pedal operation, central nervous system disease, visual acuity or visual field deficits, and cognitive impairment.

The postoperative regimen was standardized: No wrist immobilization was used, and gentle, pain-limited wrist mobilization supervised by occupational therapists began on postoperative day 1. In addition, healthy university faculty and staff and community-dwelling adults were invited to participate as controls. The inclusion criteria involved having a valid driver’s license and driving at least once per week. The exclusion criteria were the same as those for the patients.

We collected data on each participant’s sex, age, body mass index (BMI), hand dominance, and duration of driving experience. For patients, we additionally recorded the injured side, fracture pattern, and the duration from injury to surgery. Fracture pattern was classified according to the Arbeitsgemeinschaft für Osteosynthesefragen/Orthopaedic Trauma Association classification system^21^.

### DS Task

**Fig. 1:**
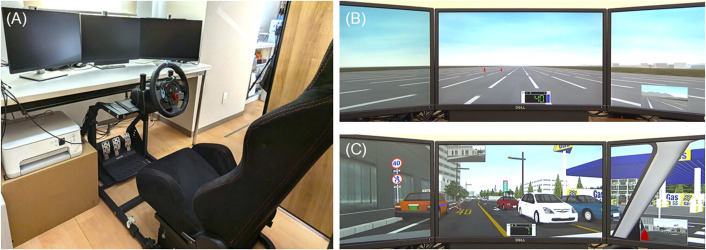
DS system. **Fig. 1-A** DS setup. **Fig. 1-B** Display of steering operation test. **Fig. 1-C** Display of hazard anticipation task. DS = driving simulator.

A Honda Safety Navi DS system (Honda Motor Co., Ltd., Tokyo, Japan) was used (Fig. 1A). The system reproduces actual driving conditions in an automatic transmission vehicle to assess driving fitness and to investigate the effects of specific functional impairments or conditions on driving safety^22-27^.

To evaluate driving performance, we administered 2 DS tasks. The Steering Operation Test requires participants to drive on an 8-lane virtual course at a maximum speed of 40 km/h and to steer between 2 pylons. Participants were instructed to pass accurately between the pylons without deviating from the lane (Fig. 1B)^28^. The Hazard Anticipation Task is designed to assess the ability to anticipate and avoid hazards in a simulated urban driving environment (Fig. 1C) and to respond appropriately^23,24^. The outcome indicators used in the 2 DS tasks, along with their definitions, are provided in Appendix I. Steering maneuvers were recorded with a digital video camera (HDR-CX470; Sony Corporation, Tokyo, Japan), and the ratio of two-handed steering was calculated as follows: two-handed steering ratio (%) = 100−([one-handed steering time with the operative hand/total task time]×100).

The starting position of both hands on the steering wheel was standardized to the traditional configuration, approximately the 10-o’clock and 2-o’clock positions for the left and right hands, respectively^29^. However, no specific instructions were provided regarding steering maneuvers after initiation of each DS task. As two-handed steering is recommended to enhance safety, particularly in situations involving sudden emergencies, high speeds, or rough road conditions^30^, participants were instructed to operate the steering wheel with both hands whenever possible.

Patients performed the DS tasks at postoperative weeks 1, 2, 3, 4, 6, and 8; healthy controls underwent a single session. At each assessment, participants performed 3 trials of the Steering Operation Test and one of the Hazard Anticipation Task.

### Wrist Functional Evaluation

Wrist pain during the DS tasks was assessed on an 11-point numerical rating scale^31^. Active ROM of the wrist and forearm was measured on the same day using a goniometer.

### Statistical Analysis

The sample size was calculated using G*Power software (version 3.1.9.7 for Windows; Heinrich Heine University, Düsseldorf, Germany)^32^ before study initiation. Based on the effect size from the pilot study^15^, the minimum required sample size was estimated at 25 per group. Furthermore, with a dropout rate of 25%^33^, 34 participants were estimated to be required.

Because the data were non-normally distributed in the Shapiro-Wilk test, nonparametric tests were used. Baseline characteristics of the patients and controls were compared using the chi-square or Mann-Whitney *U* test, as appropriate. Age and driving experience were compared between male and female individuals in the patient group using the Mann-Whitney *U* test. For the 2 DS tasks and for ROM, between-group comparisons (patients vs. controls) were performed at each postoperative time point using the Mann-Whitney *U* test. To control multiplicity due to repeated testing, P values were adjusted using the Holm method. The two-handed steering ratio and wrist pain of patients were compared across the 6 postoperative time points using a linear mixed-effects model (LMM). The primary analysis tested the main effect of postoperative time. In addition, exploratory analyses were conducted only among patients with DRFs using an LMM to examine associations between driving performance and candidate covariates. Analyses proceeded in 2 stages: a univariable LMM, followed by a multivariable model including covariates that were significant in the univariable analysis. Continuous covariates—age, BMI, driving experience, duration from injury to surgery, pain, and ROM—were entered as continuous variables without categorization. Sex, injured side, and fracture pattern were treated as categorical fixed effects. A sensitivity analysis was performed using categorized pain severity levels defined as mild (0-3), moderate (4-6), and severe (7-10)^34^.

All analyses were performed using IBM SPSS Statistics for Windows (version 29.0; IBM Corp., Armonk, NY). The level of significance was set at P < 0.05.

## Results

**Fig. 2:**
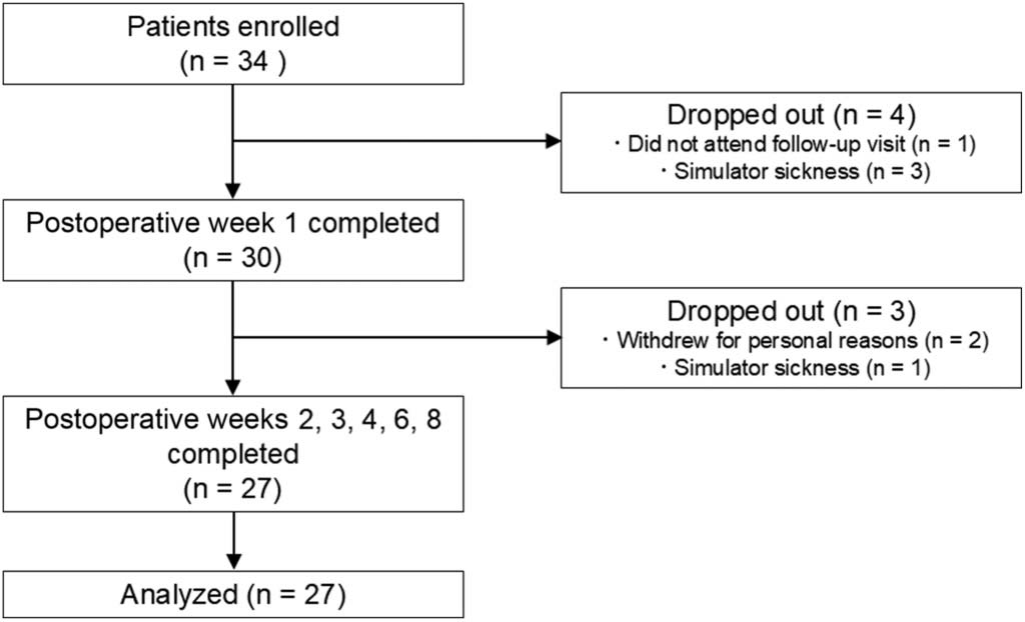
Patient enrollment flowchart.

Of the 34 patients enrolled, 27 completed all phases of the DS assessment (Fig. 2). Twenty-five healthy controls also participated; 6 required retesting on another day because of simulator sickness, but none withdrew from the study. All patients and controls were of Asian and of Japanese ethnicity. The demographic data of the patients and controls are shown in Table I. No significant between-group differences were observed. The median age in the patient group was 63.5 (interquartile range [IQR], 58.5-75.0) years for male and 63.0 (IQR, 57.5-68.0) years for female individuals, with no significant difference observed between sexes (P = 0.696). In addition, the median driving experience in the patient group was 40.0 (IQR, 32.8-51.3) years for male and 35.0 (IQR, 29.0-38.5) years for female individuals, with no significant difference observed between sexes (P = 0.333).

**TABLE I:** Demographics of Patients and Controls

Characteristic	Patients (n = 27)	Controls (n = 25)	P
Sex			
Male	8 (30%)	8 (32%)	0.853
Female	19 (70%)	17 (68%)	
Age (yr)			
Mean ± SD	63.1 ± 9.8	64.0 ± 8.5	
Median (IQR)	63.0 (57.5-68.5)	63.0 (59.0-69.0)	0.876
Range	35–80	45–79	
Body mass index (kg/m^2^)			
Mean ± SD	23.6 ± 3.7	23.0 ± 3.3	
Median (IQR)	24.3 (21.0-25.9)	22.5 (22.1-24.9)	0.492
Range	16.1–30.9	15.8–30.7	
Hand dominance			
Right	27 (100%)	25 (100%)	N/A
Left	0 (0%)	0 (0%)	
Driving experience (yr)			
Mean	36.4 ± 12.5	39.9 ± 8.2	
Median (IQR)	38.0 (29.0-42.5)	40.0 (36.0-44.0)	0.177
Range	6–63	26–56	
Injured side			
Right	9 (33%)	N/A	N/A
Left	18 (67%)	N/A	
AO/OTA classification			
A	7 (26%)	N/A	N/A
B	0 (0%)	N/A	
C	20 (74%)	N/A	
Mean	4.1 ± 4.9	N/A	
Median (IQR)	3.0 (2.0-5.0)	N/A	N/A
Range	1–27	N/A	

AO = Arbeitsgemeinschaft für Osteosynthesefragen, IQR = Interquartile range, N/A = not applicable, and OTA = Orthopaedic Trauma Association.

No significant between-group differences were observed in reaction time, left-right balance, or adaptability in the Steering Operation Test at any postoperative time point. Accuracy was significantly lower in the patients than in controls at postoperative weeks 1 and 2, whereas no significant between-group differences were observed from week 3 onward (Table II). In the Hazard Anticipation Task, no significant between-group differences were observed for any indicators (Table II). In addition, the two-handed steering ratio remained high from postoperative week 1 onward, and no significant main effect of time was observed (P = 0.200; Appendix II).

**TABLE II:** Results of Each Performance Indicator in the Driving Simulator Tasks

Performance indicator	Controls	Postoperative wk											
		1	P	2	P	3	P	4	P	6	P	8	P
Steering Operation Test													
Reaction time (second)													
Mean ± SD	2.0 ± 0.2	2.0 ± 0.1	0.840	2.0 ± 0.1	1.000	2.0 ± 0.1	1.000	2.0 ± 0.1	1.000	2.0 ± 0.1	1.000	2.0 ± 0.1	1.000
Median (IQR)	2.0 (1.9-2.1)	2.0 (1.9-2.0)		2.0 (1.9-2.1)		2.0 (1.9-2.1)		2.0 (2.0-2.1)		2.0 (1.9-2.2)		2.0 (1.9-2.1)	
Accuracy (%)													
Mean ± SD	67.3 ± 20.5	49.9 ± 20.5	0.006*	55.6 ± 19.1	0.040*	58.9 ± 21.5	0.208	60.3 ± 23.1	0.210	63.3 ± 21.4	0.574	66.5 ± 22.1	0.685
Median (IQR)	57.3 (50.0-92.7)	44.8 (36.5-51.6)		46.9 (43.2-63.3)		49.0 (43.8-70.3)		51.6 (44.8-81.5)		52.1 (47.9-88.0)		57.3 (47.9-88.5)	
Left-right balance (%)													
Mean ± SD	−14.6 ± 32.3	−48.4 ± 46.4	0.138	−36.2 ± 47.6	1.000	−37.3 ± 55.1	1.000	−32.8 ± 51.9	1.000	−24.5 ± 48.0	1.000	−20.6 ± 50.0	1.000
Median (IQR)	−9.6 (−26.0 to 7.7)	−59.8 (−100 to 5.4)		−19.6 (−91.5 to 6.9)		3.2 (−100 to 13.4)		−1.4 (−100 to 12.2)		2.0 (−61.5 to 11.9)		5.7 (−80.6 to 12.4)	
Adaptability (second)													
Mean ± SD	0.0 ± 0.1	−0.1 ± 0.2	1.000	−0.1 ± 0.2	1.000	−0.1 ± 0.1	1.000	−0.1 ± 0.1	1.000	0.0 ± 0.1	1.000	0.0 ± 0.1	1.000
Median (IQR)	−0.1 (−0.1 to 0.0)	−0.1 (−0.2 to 0.0)		−0.1 (−0.1 to 0.0)		−0.1 (−0.1 to 0.0)		−0.1 (−0.1 to 0.0)		0.0 (−0.1 to 0.0)		0.0 (−0.1 to 0.0)	
Hazard Anticipation Task													
Maximum steering angle (radian)													
Mean ± SD	11.6 ± 2.0	10.8 ± 2.2	0.535	11.2 ± 1.8	0.828	10.9 ± 2.5	0.656	10.9 ± 2.1	0.656	10.6 ± 1.9	0.420	11.0 ± 2.1	0.656
Median (IQR)	11.3 (10.4-12.2)	10.5 (9.6-12.0)		11.6 (10.0-12.5)		9.8 (9.2-11.9)		10.8 (9.3-12.4)		10.4 (9.5-11.5)		10.4 (9.1-13.0)	
Collision (number)													
Mean ± SD	1.2 ± 1.2	1.3 ± 2.3	1.000	1.0 ± 1.0	1.000	1.7 ± 1.4	1.000	1.2 ± 1.4	1.000	0.8 ± 0.7	1.000	1.4 ± 2.0	1.000
Median (IQR)	1.0 (0.0-2.0)	1.0 (0.0-1.0)		1.0 (0.0-2.0)		2.0 (0.0-2.5)		0.0 (0.0-2.0)		1.0 (0.0-1.0)		1.0 (0.0-2.0)	
Percentage of distance speeding (%)													
Mean ± SD	4.8 ± 7.8	4.1 ± 5.9	1.000	5.5 ± 6.1	1.000	6.0 ± 6.9	1.000	4.7 ± 5.7	1.000	4.1 ± 6.7	1.000	4.9 ± 6.9	1.000
Median (IQR)	0.0 (0.0-6.0)	0.9 (0.0-6.7)		3.9 (0.0-8.6)		4.1 (0.0-7.4)		2.6 (0.0-6.5)		0.0 (0.0-5.5)		2.1 (0.0-7.1)	
Average excess speed (km/h)													
Mean ± SD	4.5 ± 6.3	4.9 ± 5.7	1.000	6.7 ± 5.8	0.450	6.5 ± 5.6	0.505	5.4 ± 5.8	1.000	4.0 ± 4.8	1.000	5.1 ± 5.5	1.000
Median (IQR)	0.0 (0.0-7.7)	3.0 (0.0-8.6)		7.7 (0.0-10.9)		7.1 (0.0-9.4)		6.0 (0.0-9.7)		0.0 (0.0-7.2)		5.6 (0.0-8.4)	
Average turning speed at intersections (km/h)													
Mean ± SD	13.3 ± 4.9	15.9 ± 4.1	0.240	15.0 ± 6.6	1.000	15.3 ± 5.7	0.805	14.1 ± 5.8	1.000	14.5 ± 5.7	0.912	15.4 ± 6.4	0.912
Median (IQR)	13.0 (9.3-16.1)	14.4 (12.7-19.5)		13.0 (10.1-17.4)		13.9 (12.8-18.3)		13.3 (11.6-18.3)		14.6 (11.5-17.0)		15.0 (12.3-18.1)	
Turn signal errors (number)													
Mean ± SD	3.8 ± 2.3	3.2 ± 1.9	1.000	3.0 ± 1.6	1.000	3.4 ± 1.6	1.000	3.1 ± 1.6	1.000	3.4 ± 2.1	1.000	3.2 ± 1.4	1.000
Median (IQR)	3.0 (3.0-5.0)	2.5 (2.0-5.0)		2.5 (2.0-4.0)		3.0 (2.5-4.0)		3.0 (2.0-3.8)		3.0 (2.0-4.0)		3.0 (2.0-4.0)	

* Indicates a significant difference compared with controls at an adjusted p value < 0.05 (two-sided Mann-Whitney *U* tests; Holm correction for multiple comparisons). IQR = interquartile range.

Median wrist pain of patients during driving decreased significantly over the postoperative course (main effect of time, P < 0.001; Appendix III). The median ROM of the wrist and forearm increased over the postoperative course; at 8 weeks, median forearm supination ROM recovered to the level of the unaffected side, whereas other ROM values remained significantly lower than those of the unaffected side (Appendix IV).

In the univariable LMM, accuracy on the Steering Operation Test, which differed significantly from that of controls, was negatively associated with female sex and older age. Positive associations were observed for wrist extension, ulnar deviation, and forearm pronation (Table III). Pain was not significantly associated with Steering Operation Test accuracy, either when treated as a continuous variable or when categorized into 3 levels (overall effect for the 3-level categorical analysis, P = 0.606). In the multivariable LMM, female sex and older age remained significantly negatively associated with Steering Operation Test accuracy, whereas forearm pronation remained significantly positively associated (Table III).

**TABLE III:** Associations Between Covariates and Steering Accuracy in a Linear Mixed-Effects Model

Covariates	Univariable			Multivariable		
	Estimated Effect (β)	95% CI	P	Estimated Effect (β)	95% CI	P
Sex (female)	−18.598	−31.184 to −6.012	0.005*	−15.624	−26.760 to −4.488	0.008†
Age	−1.004	−1.584 to −0.425	0.001*	−0.940	−1.466 to −0.413	0.001†
BMI	1.620	−0.105 to 3.345	0.064			
Driving experience	−0.497	−1.008 to 0.014	0.056			
Injured side (right side)	9.936	−3.712 to 23.585	0.146			
AO/OTA classification (Type A)	3.331	−11.669 to 18.330	0.651			
Injury-surgery interval	−0.841	−2.165 to 0.484	0.202			
Pain	−0.261	−0.857 to 0.334	0.384			
ROM						
Flexion	0.078	−0.020 to 0.176	0.117			
Extension	0.167	0.056 to 0.278	0.004*	0.112	−0.001 to 0.224	0.052
Radial deviation	0.080	−0.074 to 0.234	0.295			
Ulnar deviation	0.204	0.058 to 0.350	0.008*	0.142	−0.011 to 0.296	0.069
Pronation	0.172	0.068 to 0.276	0.002*	0.160	0.057 to 0.263	0.003†
Supination	0.020	−0.104 to 0.145	0.745			

For categorical covariates, β represents the difference in steering accuracy for the category indicated in parentheses relative to the reference category (not shown). For continuous covariates, β represents the change in steering accuracy per 1-unit increase in the covariate.

* Significant association with steering accuracy in the univariable linear mixed-effects model.

† Significant association with steering accuracy in the multivariable linear mixed-effects model.

AO = Arbeitsgemeinschaft für Osteosynthesefragen, BMI = body mass index, CI = confidence interval, OTA = Orthopaedic Trauma Association, and ROM = range of motion.

## Discussion

Our study highlights that, by postoperative week 3, the driving performance of patients with DRFs treated with VLP no longer differed significantly from that of healthy controls. Therefore, postoperative week 3 could serve as a reference point when considering return to driving. Patients resumed driving at a mean of 15.8 days after VLP^35^, suggesting that patient-initiated return to driving may occur before adequate driving performance has been regained. Our results of 3 weeks may serve as a useful reference to help physicians provide evidence-based guidance to postoperative patients on the safe timing of return to driving.

Previous studies have reported wide variability in the recommended time to resume driving after VLP^4,14,15^. Certain patients could safely resume driving within 3 weeks postoperatively^4^. However, several driving-simulator performance-related differences between patients with DRFs and healthy controls were no longer apparent by approximately postoperative week 7^15^. Furthermore, Tan et al. reported that patients passed an on-road driving assessment at postoperative week 8^14^. In these studies, postoperative wrist immobilization was used, with variation in both the method of application and the duration, which may delay recovery of wrist function and, consequently, driving performance. We adopted a postoperative regimen without external immobilization, and all patients underwent driving assessments without immobilization from an early stage. We believe this yielded a recommended time for resuming driving that was similar to that reported by Jones et al.^4^ who suggested the earliest timing among previous studies.

Steering accuracy was significantly reduced at postoperative weeks 1 and 2, but did not differ significantly from that of control results by week 3. By contrast, reaction time, left-right balance, and adaptability did not differ significantly from those of healthy controls from postoperative week 1 onward, possibly reflecting differences in the driving demands assessed by each indicator. These measures primarily reflect the temporal response required for lane changes and may therefore recover relatively early. By contrast, steering accuracy for passing between pylons reflects spatial precision and likely requires more precise two-handed control, placing greater demands on wrist function on the operated side. The improvement in steering accuracy, with no significant difference from healthy controls observed by postoperative week 3, might be attributable to the higher contribution of the operative hand to steering as wrist function recovered through pain reduction and increased ROM. Our median driving-pain score of 1.5 at postoperative week 3 was comparable with that reported for the initial-pass group by Jones et al^4^. At 3 weeks, wrist and forearm ROM were broadly consistent with those required for steering as previously reported^36^ (Appendix V). Safe driving requires that patients have normal motor function and adequate joint mobility^37^. Recovery of wrist function after VLP is gradual; by 1 year postoperatively, wrist ROM reaches at least 90% of that of the unaffected side^38,39^, and the side-to-side difference becomes nonsignificant^40^. By contrast, patients managed without postoperative immobilization and with early wrist mobilization show adequate ROM required for steering by postoperative week 3.

In the Hazard Anticipation Task, no significant differences were observed between patients and healthy controls in any indicator from postoperative week 1 onward. Furthermore, the maximum steering angle was similar to that of controls even in the early postoperative period, and the observed two-handed steering ratio remained high from week 1. Although two-handed steering is recommended from a safety perspective^30^, our findings suggest that the operated hand may have contributed to two-handed steering as early as postoperative week 1.

Badger et al. reported that patients who underwent arthroscopic rotator cuff repair could safely resume driving as early as 2 weeks postoperatively based on instrumented on-road assessments of driving kinematics and behavioral performance^41^. At that time, driving was performed with the arm immobilized in a sling, and the authors suggested that heightened caution arising from awareness of physical limitations may have contributed to safety^41^. By contrast, during the early postoperative period in our study, no significant differences were observed between patients and healthy controls in the percentages of distance speeding, average excess speed, or average turning speed at intersections. Despite limited evidence of unusually cautious driving, the number of collisions did not differ significantly. This may be attributable to the fact that, as postoperative external immobilization was not applied, the operated hand was able to participate to a certain extent in steering operation. Nevertheless, in light of the observed decline in steering accuracy at postoperative weeks 1 and 2, and considering the findings as a whole, it is reasonable to consider return to driving from postoperative week 3.

In our multivariable analyses, female sex and older age were associated with lower steering accuracy, whereas greater forearm pronation ROM was associated with higher accuracy. These findings are consistent with the report by Poiset et al.^35^, which identified female sex and older age as independent predictors of delayed return to driving after VLP. An epidemiologic study has shown that, across all age groups, female individuals drive fewer miles per year on average than male individuals^42^. In this study, although no significant sex-based difference in years of driving experience was observed in the patient group, women may have had lower annual driving mileage and, therefore, potentially lower driving proficiency. In addition, a DS study suggested that female individuals tend to have shorter perception times but longer movement times^43^. Movement time is thought to be influenced primarily by physical factors, such as muscle strength, and the relative advantage observed in male individuals has been attributed in part to greater muscle strength^43,44^. Collectively, these factors may have contributed to the lower steering accuracy observed among women in this study. With respect to forearm pronation, biomechanical studies have reported that palmar contact is the primary contributor during steering and that forearm pronation-supination is important for achieving such contact^36,45^. Forearm pronation is also responsible for rotating the steering wheel toward the contralateral side^36^ and may therefore contribute strongly to steering accuracy.

This study has some limitations. First, the sample size was relatively small, and 67% of the patients sustained injuries on the nondominant side. In addition, the median participant age was in the 60s, and all participants were right-handed. Therefore, the study population may not have fully reflected clinical diversity in patient characteristics and clinical presentation. Accordingly, caution is warranted when generalizing our findings to younger individuals, left-hand–dominant individuals, and patients with dominant-side injuries. The a priori power analysis was based on a 2-group comparison, and the univariable and multivariable analyses should therefore be considered exploratory. Accordingly, given the sample size constraints, these analyses may have been subject to type II error and may not have fully detected relevant associated factors. Furthermore, because potential interactions were not modeled, they were not formally evaluated. Second, our findings were obtained from a DS that modeled an automatic transmission vehicle and thus may not be directly generalizable to manual transmission vehicles, which impose relatively greater upper-extremity demands^46^. Third, on-road driving involves a series of tasks, including opening and closing the door, fastening and unfastening the seatbelt, and operating the ignition and shift lever^12^, which were not assessed in this simulator-based study. Accordingly, the present findings should be interpreted as reflecting recovery of simulator-based driving performance under controlled conditions, rather than overall on-road driving competence. Fourth, the DS was configured for a right-hand–drive vehicle and left-side traffic; therefore, caution is warranted when extrapolating our results to left-hand–drive vehicle and right-side traffic environments. Therefore, our findings should be generalized cautiously to younger individuals, left-hand–dominant individuals, and patients with dominant-side injuries. Future studies should examine the relationship between simulator-based outcomes and on-road driving performance and confirm generalizability across more diverse driving conditions and patient populations.

## Conclusion

Among patients with DRFs treated with VLP without postoperative wrist immobilization, longitudinal assessment over the first 8 postoperative weeks revealed that simulator-based driving performance did not differ significantly from that of healthy controls by postoperative week 3. Therefore, postoperative week 3 might serve as a reference point when considering return to driving. As a female sex, older age, and insufficient recovery of forearm pronation ROM might adversely affect steering accuracy, a graded and cautious approach to return to driving should be considered.

## Appendix

Supporting material provided by the authors is posted with the online version of this article as a data supplement at jbjs.org (http://links.lww.com/JBJSOA/B251). This content was not copyedited or verified by JBJS.
